# Absence of disparities in anthropometric measures among Chilean indigenous and non-indigenous newborns

**DOI:** 10.1186/1471-2458-10-392

**Published:** 2010-07-03

**Authors:** Hugo Amigo, Patricia Bustos, Jay S Kaufman

**Affiliations:** 1 Department of Nutrition, Faculty of Medicine, University of Chile, Chile; 2 Department of Epidemiology, Biostatistics and Occupational Health, Faculty of Medicine, McGill University, Canada

## Abstract

**Background:**

Studies throughout North America and Europe have documented adverse perinatal outcomes for racial/ethnic minorities. Nonetheless, the contrast in newborn characteristics between indigenous and non-indigenous populations in Latin America has been poorly characterized. This is due to many challenges, including a lack of vital registration information on ethnicity. The objective of this study was to analyze trends in anthropometric measures at birth in Chilean indigenous (Mapuche) and non-indigenous children over a 5-year period.

**Methods:**

We examined weight and length at birth using information available through a national data base of all birth records for the years 2000 through 2004 (n = 1,166.513). Newborns were classified ethnically according to the origins of the parents' last names.

**Result:**

The average birthweight was stable over the 5 year period with variations of less than 20 g in each group, and with mean values trivially higher in indigenous newborns. The proportion weighing less than 2500 g at birth increased modestly from 5.2% to 5.6% in non-indigenous newborns whereas the indigenous births remained constant at 5.2%. In multiple regression analyses, adjusting flexibly for gestational age and maternal characteristics, the occurrence of an indigenous surname added only 14 g to an average infant's birthweight while holding other factors constant. Results for length at birth were similar, and adjusted time trend variation in both outcomes was trivially small after adjustment. Anthropometric indexes at birth in Chile are quite favorable by international standards.

**Conclusion:**

There is only a trivial degree of ethnic disparity in these values, in contrast to conditions for ethnic minorities in other countries. Moreover, these values remained roughly constant over the 5 years of observation in this study.

## Background

Characteristics of the newborn child reflect the complex web of intrauterine conditions and their interactions with the genetic potential of the individual. It is the multifaceted determination of these measures that makes them so useful not only as prognostic indicators for the child's health and development, but also as a robust index of social conditions and maternal health. Recently, an additional interest in these measures has centered around the possible association between a deficit in intrauterine growth and the elevation of risk for chronic diseases later in adult life [1,2].

Birthweight has long served as one of the most important indicators of maternal-child health, especially in the simple dichotomy of "low birth weight", often defined as birthweight less than 2500 g. The proportion of such births has been a mainstay of international comparisons for decades, and generally shows less favorable distribution in less developed countries[3]. Nonetheless, some questions remain, such as the counterintuitive existence of countries in Africa with very high rates of poverty and yet with birthweight statistics that are not as dire as those seen in similarly poor countries in Southeast Asia [3,4].

Birthweight has been considered in the public health and clinical literatures in many transformations, including the comparison of means or medians of the continuous measure, as well as the proportion below a cutpoint such as 2500 g. Tail proportions capture something of the spread of the distribution, while a mean only reveals where the distribution is centered, and thus the different indices reveal different aspects of population health. These considerations have been discussed in the work of Wilcox [5] and others, with the general consensus emerging that low birthweight has profound implications for rates of infant mortality, whereas measures of central tendency indicate population health and social conditions, including the effects of reproductive technologies and medical interventions.

In less developed nations, low birthweight may more commonly arise from intrauterine growth retardation (IUGR), whereas in developed nations it may arise more commonly because of shorter gestational ages (i.e. preterm birth) [6]. Recent trends in developed nations indicate a possible increase in the proportion of low birthweight deliveries in contrast to decades of improvement [7]. This observation is consistent with a wide variety of explanations, including the effect of reproductive technologies on multiple gestations, the overutilization of cesarean delivery and changes in demographic characteristics of mothers, such as age and obesity.

Chile is a country with an intermediate level of development and has experienced this last decade a rapid epidemiological transition, with major shifts in population into urban areas and into formal sector employment and supervised pregnancy. Overall, there has been rapid increase in standard of living for the population in aggregate. Furthermore, there has been a longstanding program of interventions targeted to mothers and children. These structural interventions have been characterized by an integrated vision of health and the life cycle with a priority for health care in ambulatory and community settings. The emphasis was placed on an interwoven concept of health and health service organizations and a continuing process of evaluation toward permanent improvement in the quality of the services [8]. At the same time that maternal and child health was prioritized, however, Chilean society overall has been characterized by severe inequalities in wealth and health [9,10].

As in most of Latin American, one of the most highly disadvantaged groups in Chilean society has remained the indigenous inhabitants. The Mapuche, also called Araucanos, constitute the most important ethnic group in the nation in both population size and degree of cultural influence. Nevertheless, official statistics on health disparities between Mapuche and non indigenous populations are generally unavailable, due in part to the failure to collect health information categorized by ethnicity.

In the Latin American context, Chile provides an ideal setting in which to examine birth outcomes given a modern and well-functioning system of vital registration that is essentially universal and is maintained electronically. Over 98% of labors are performed at specialized medical centers under the care of clinical nurse midwives [11] and an extensive program of prenatal control has existed for decades. Furthermore, Chile represents a rich opportunity to consider health disparities by categories of ethnicity (i.e. aboriginal compared to European) since the majority of the culturally-identified indigenous population belongs to a single ethnic grouping of profound historical and social significance: the Mapuche. In this paper we will examine birth outcome measures comparing non indigenous to those with Mapuche identity, considering trends in these disparities over time, for the entire nation of Chile for the years 2000 to 2004.

## Methods

The universe of interest was defined as all live singleton births in Chile for the years 2000 through 2004. Data on all live births were collected, recorded by the National Institute of Statistics (INE) and the Civil Registration Office in the location where the delivery occurred. Numerous quantities are captured in the vital registration system, including weight in grams, length in centimeters, clinical estimate of gestational age at delivery and the reported level of education of the mother. Weight and length at birth are recorded by clinical nurse midwives trained to obtain these measures according to norms established and maintained by the Chilean Ministry of Health [12]. These specifications describe the type of equipment to be used and its use, and the techniques are illustrated and explained in written instructions. The gestational age of the newborn was calculated considering the date of the last menstrual period, physical exam of the newborn and ultrasound when clinically indicated.

Ethnicity is not collected in Chilean vital statistics, and so in order to analyze the data by Mapuche or Non-Mapuche subgroups, it was necessary to develop an algorithm for making this distinction. This was accomplished by compiling an extensive data base of more than 7,000 Mapuche surnames [13]. The electronic files of birth records were then matched to this data base by means of a computer program that screened the surnames of both parents of each newborn. If either parent had a recognizable Mapuche surname, the child was classified as Mapuche, making the designation more sensitive and less specific than requiring both parents to have Mapuche surnames.

The mother's years of completed schooling were considered to form a socioeconomic status proxy. This information is provided by the mother when registering the baby at the Civil Registration Office. The variable was categorized in the descriptive analysis into the following groups: elementary education (up to 8 years of school), secondary school (those who had between 8 to 12 years) and those who reported having further university-level education (more than 12 years of instruction). In multiple regression analyses, the variable was used in continuous form (i.e. in units of completed years of education).

In descriptive analyses, birthweight was divided into five categories: <1500 g, 1500 g to 2499 g, 2500 g to 2999 g, 3000 g to 3999 g and those who weighed over 4000 g at birth. To analyze the time trends for birthweight and length at birth in indigenous and non indigenous newborns, multiple linear regression models were fit, with birthweight (g) and length (cm) as the dependent variables and with socioeconomic and demographic factors (rural area, age and years schooling of the mothers, sex and gestational age of the newborn) as control variables. A variable for calendar year from 2000 to 2004 was included to assess time variability. Linearity assumptions were relaxed for continuous variables (gestational age and mother's age) by adding quadratic terms to the model.

This study was approved by the Ethics Committee of the Faculty of Medicine of the University of Chile and had the authorization of the Statistics Division of the Ministry of Health of Chile.

## Results

**Figure 1:**
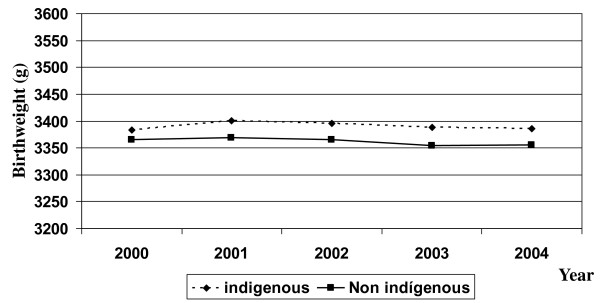
**Birthweight by year in indigenous and non indigenous Chilean newborns**.

**Figure 2:**
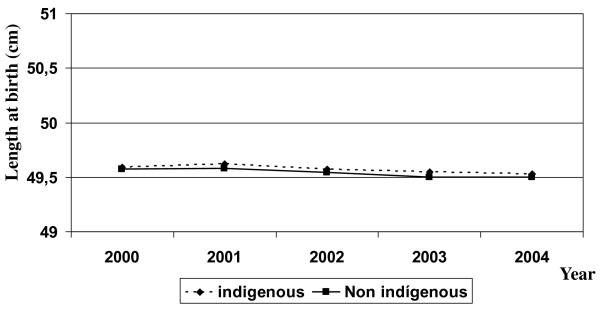
**Length at birth by year in indigenous and non indigenous Chilean newborns**.

A total of 1,166,513 live births were recorded, representing the universe of Chilean births occurring from January 1 2000 to December 31 2004. Of these, 1,145,752 were singleton newborns analyzed in this study. In the non-indigenous population there was a small but consistent decrease in the number of births that formed a decline of 2.7% over the study period. In the indigenous population, in contrast, the birth rate was approximately stable throughout the period of observation. The excess proportion of male births also remained relatively stable, fluctuating between 102.9 and 105.5% of the female births. The proportion of multiple births did not exceed 2% and was higher among the non-indigenous infants. The proportion of preterm births (born before 37 weeks of gestation) increased slightly over the interval, especially in non-indigenous children, reaching figures of 6.0% in the year 2000 and 6.9% the year 2004. In relation to the mother's years of schooling, the pattern was different between ethnic groups; in the indigenous the proportion of mothers with university education did not exceed 10% but with a modest increase over time, whereas among the non-indigenous this proportion was close to 20%, also with an upward trend (Table 1). Mean birthweight was also relatively stable in the period considered, with variations that do not exceed 20 g in either ethnic group. In each of the 5 years studied, the mean birthweights were slightly higher in the indigenous newborns. (Figure 1). With respect to length at birth, similar patterns were observed, as shown in Figure 2.

**Table 1:** Socio-demographic characteristics of the newborn according to year of birth in indigenous and non indigenous newborns

Year	Total births	Mothers age (years)		Male/Female	Multiple births	Weeks of gestation	Prematurity*	Mothers schooling (%)			
	(n)	Mean	(SD)	×100	(%)	(mean)	(%)	University	Secondary	Basic	None
2000											
Indigenous	16,726	26.2	(6.6)	104.5	1.2	38.8	5.9	7.6	51.1	40.6	0.6
Non indigenous	221,184	26.9	(6.6)	105.5	1.7	38.7	6.0	19.2	59.2	21.2	0.2
2001											
Indigenous	17,043	26.6	(6.7)	103.7	1.6	38.8	5.4	6.9	50.9	41.6	0.6
Non indigenous	220,060	27.0	(6.7)	103.6	1.9	38.7	6.0	18.0	59.1	22.4	0.2
2002											
Indigenous	14,594	26.7	(6.7)	103.6	1.5	38.8	5.8	7.6	50.4	41.4	0.6
Non indigenous	216,531	27.1	(6.7)	104.3	1.9	38.7	6.3	19.0	59.4	21.2	0.2
2003											
Indigenous	15,030	26.9	(6.7)	104.6	1.7	38.8	6.5	8.5	51.9	39.1	0.5
Non indigenous	214,993	27.2	(6.7)	104.4	1.8	38.7	6.5	20.6	59.0	20.2	0.2
2004											
Indigenous	15,273	26.9	(6.9)	102.9	1.4	38.8	6.1	9.0	52.7	37.9	0.4
Non indigenous	215,079	27.3	(6.7)	104.0	1.8	38.6	6.9	22.0	58.7	19.1	0.2

Total											
Indigenous	78,666	26.7	(6.7)	103.7	1.5	38.8	5.9	7.6	51.1	40.7	0.6
Non indigenous	1.087,847	27.1	(6.7)	104.5	1.8	38.7	6.4	19.3	59.2	21.2	0.2

* births prior to 37 weeks of gestation

The percentage of newborns with low birth weight (<2500 g) was similarly stable over the 5 year interval, increasing modestly from 5.25% to 5.63% in non-indigenous newborns and remaining roughly constant in the indigenous group with slightly lower values. The indigenous newborns showed the highest proportion with birthweight over 4000 g, one percent higher than non-indigenous newborns in all the years studied (Table 2).

**Table 2:** Percentage of singleton births in categories of birthweight, 2000 to 2004

Weight	<1500 g		1500-2499 g		2500-2999 g		3000-3999 g		≥4000 g	
**Years**	**Non** **indigenous**	**Indigenous**	**Non** **indigenous**	**Indigenous**	**Non** **indigenous**	**Indigenous**	**Non** **indigenous**	**Indigenous**	**Non** **indigenous**	**Indigenous**

2000	0.88	0.83	4.37	4.38	15.21	14.27	69.82	69.57	9.62	10.80
2001	0.86	0.82	4.39	4.10	14.96	13.40	69.85	70.35	9.63	11.05
2002	0.90	0.76	4.48	4.33	15.06	13.75	69.84	70.35	9.43	11.19
2003	0.94	0.92	4.46	4.39	15.13	13.90	70.72	70.01	8.60	10.55
2004	0.92	0.82	4.71	4.36	15.37	14.01	69.66	69.62	9.11	10.75

Another approach to analyzing the disparities between ethnic groups and over time is through multiple regression, as this allows for conditional estimates (i.e. the effect of one covariate while holding another variable constant). In fully adjusted models, having an indigenous surname increased birthweight by 12 g holding all other factors constant (Table 3). The secular change over the 5 year period after adjustment was an increase of about 2 g per year. The R^2 ^for the model is 0.39. In the case of length at birth, there was also a detectable effect of the year of observation, although trivial in magnitude (0.05 mm per year). Being indigenous decreased the length at birth by 0.17 mm after multivariable adjustment, a smaller effect than living in a rural area (0.11 mm). The R^2 ^for the model is 0.46.

**Table 3:** Adjusted* effect of the time (trend) and ethnic group on birthweight (g) and length at birth (mm) of Chilean newborns, 2000-2004

	Change in birthweight (g)	95% confidence interval	Change in birth length (mm)	95% confidence interval
Tendency (years) **	1.99	1.45, 2.52	0.05	0.03, 0.08
Indigenous surname	12.45	9.39, 15.51	-0.17	-0.31, -0.03
R-squared	0.39		0.46	

* adjusted for gestational age, gestational age squared, maternal age, maternal age squared, mother's years of education, rural/urban residence, and sex of the child.

** Tendency: annual change

## Discussion

An analysis of over a million births in a middle-income nation reveals a favorable distribution of anthropometric measures at birth, independently of ethnic origin. This is reflected in the modest prevalence of children with low weight at birth, for which indigenous children have an even lower proportion (although trivially so). The time trends in these anthropometric variables during the 5 year period of observation in this study is roughly stable, with a modest but detectable increase of weight at birth observed after adjustment for gestational age and other variables.

The absence of an ethnic disparity in these outcomes is surprising because Chileans with Mapuche identity have substantial socioeconomic disadvantages, and notable health disparities for many other outcomes [14,15]. Nonetheless, a well-established and actively supported prenatal control program appears to keep social disparities in reproductive outcomes quite limited in Chile, despite a wide gulf between rich and poor in the society [16].

These results suggest that with respect to the usual indicators of reproductive health, Chile behaves much more like a developed country. Birth outcome statistics in this study are very good in comparison with the rest of the region, and approach patterns typical of North America and Europe [17,18], resulting from a potentially wide variety of factors, including changes in patterns of obstetric care as well as improved general nutrition and access to prenatal care [19,20]. Nonetheless, while developed nations experience low rates overall of insufficient intrauterine growth, they can also have dramatic disparities by social class or by ethnicity. In this study, a socially disadvantaged group was not seen to suffer from any substantially higher risk of insufficient fetal growth, which is an important indication that such disparities are avoidable.

Better conditions of pregnancy in Chile may be explained through the rapid overall improvement of socioeconomic conditions represented, for instance, in a rapidly increased gross national product (GNP) and a similarly precipitous decrease in levels of poverty. Chile's GNP grew by a remarkable 7% per year in the 1990s, although economic growth has moderated in the last few years, as in most of the world economy[21]. Concomitantly, Chile witnessed a profound decrease in levels of poverty and homelessness, which fell by 60% over a period of 20 years [22]. It is entirely plausible that these broad economic changes affected many individuals through improvement in mothers' nutritional status that may be inferred, for example, by an observed reduction in underweight pregnant women from 25 to 15% between 1987 and 1993 [23].

The somewhat surprising result that the indigenous population does not suffer from adverse anthropometric conditions at birth suggests that by the first decade of the 21st Century, the kind of deprivation that affects so much of the indigenous population of Latin American is conquered in Chile, at least to the extent that pregnant women appear to have sufficient resources and pregnancies that are on average as healthy as those in the majority population. While vast inequalities in access to health and resources still exist in Chile, it seems that these are not evident in pregnancy, and that public health attention can be shifted to later life conditions. This situation may be peculiar to the Chilean indigenous population, although there is some published evidence to suggest that in other places on the continent also, malnutrition initiates after weaning [24,25].

The fact that more than 10% of the country's population weighs more than 4000 g at birth and that this proportion is actually higher in indigenous newborns may be considered a public health alert, since various studies are now reporting high prevalence of excess weight in school children. The trajectories of weight gain connecting birthweight to adult weight are not yet clear, but this could be quite an important fact in a country like Chile that is experiencing such an explosive growth in excess weight in almost all age groups [26].

Length at birth has been described as a predictor of "stunting" at subsequent ages [27] but for operational reasons this index has not generally been included in routine records at the level of health centers and the systems of nutritional surveillance. It has been confirmed in the current paper that length at birth in Chile does not reach the levels recommended by international standards. This may therefore contribute to explaining the prevalence of "stunting" in the population that is higher than would be expected from Chile's overall health and social status indicators. This outcome is especially prominent in populations of low socioeconomic position [28].

Many hypotheses are available to make sense of these patterns. For example, the proportion of multiple births has been stable, while the duration of pregnancy has decreased slightly, which may reflect the situation that newborns from more advantaged social classes, in which non-indigenous parents predominate, have shorter pregnancies. This confounding by social class would serve to balance out the averages of weight and length to erase a true disparity that exists within strata, although control for mother's education did not have a profound influence on these results. Of particular note when considering socioeconomic confounding is the fact that Chile currently has one of the highest percentages of caesareans in the region, approaching up to 40% of births, which is almost the double that seen in many other Latin American countries. This procedure now occurs in one out of 3 labors in public hospitals and in 60% in private ones, which can also introduce important confounding due to the truncation of the gestational period [29].

The continuing modest trend toward heavier births in Chile is potentially related to the disappearance of gaps between social groups, and is a phenomenon that has not been well described in Latin America. Other changes have occurred in relation to pregnancy and childbirth in Latin America which affect anthropometric measures in newborns, and are surely part of the complex mix in the Chilean situation as well. These include not only changes in social conditions such as income, poverty, nutrition and mothers' employment, but also an increase in the average intergestational interval, increases in prevalence of gestational diabetes, and decreases in the prevalence of smoking among pregnant women [30,31]. Additionally, the absence of disparities at the Chilean context may also reflect an investment in pregnancy care including adherence to prenatal care and specialized nutritional intervention programs for mothers in risk.

One of the limitations of this study is the use of the gestational age variable, information that was obtained from the birth certificate, which is an amalgam of different pieces of information (i.e., date of last menstrual period, neonatal examination and ultrasound). The algorithm for combining these pieces of information varies from one place to another, and it is quite likely that health units that assist rural populations do not have the same availability of equipment as those located in areas of higher demand or centers attended by higher income urban women. Although there exists no "gold standard" for assessment of gestational age, the use of several pieces of data in combination is supposed to be a better approximation. Nonetheless, this is a source of some uncertainty in our data set, and a possible target for sensitivity analysis or other corrections.

This study was conducted on the universe of those born alive in the country, and data have gone through quality control. The fact that there was no sampling suggests that there is no need to rely on inferential statistics such as p-values or confidence intervals. The latter are provided to demonstrate the great precision of the estimates, but in fact they do not have any formal interpretation because the entire population was included in the study [32]. This advantage, relying on vital registration data in a country with essentially universal coverage, carries with it the attendant disadvantage of secondary data. Although data quality is known to be high in Chile, the data are collected by a large number of individuals in a wide variety of settings, leading to some inevitable variability in method or quality. For example, although the population delivering outside of medical supervision is very low (less than 2%), it is notably higher among those who register their births late (5%) and the latter situation may apply differentially by ethnic group status, since many Mapuche live in isolated rural communities.

Although we have great precision to detect small variations in the measures obtained, this does not imply that such small differences necessarily have a clinical relevance. Nonetheless, our work is engaged from a population perspective. While there is variability in every population group, the mean birthweight for the Chilean indigenous population of newborns differs only trivially from the mean birthweight for the non-indigenous population. In this respect, disparities in this outcome, one of the most universally acknowledged markers of population health, have largely disappeared.

## Conclusion

This study has reported the absence of any important differences in anthropometric measures at birth between ethnic groups for a 5-year period among singleton live births in Chile. The data also reveal a low prevalence of children with low weight at birth. The time trends in these anthropometric variables is relatively stable, with a modest but detectable increase of weight at birth, conditional on gestational age and other covariates. The absence of an ethnic disparity in these outcomes is surprising because Chileans with indigenous background have documented socioeconomic disadvantages along with resulting health disparities for other outcomes.

## Competing interests

The authors declare that they have no competing interests.

## Authors' contributions

HA and PB were in charge of the planning, collection of the data, analysis and writing the paper. JK was also responsible by the analysis, statistic advice, and writing, editing and approving the final version of the submitted manuscript.

## Pre-publication history

The pre-publication history for this paper can be accessed here:

http://www.biomedcentral.com/1471-2458/10/392/prepub
